# A novel lily anther-specific gene encodes adhesin-like proteins associated with exine formation during anther development

**DOI:** 10.1093/jxb/eru051

**Published:** 2014-03-03

**Authors:** Ming-Che Liu, Cheng-Shou Yang, Fang-Ling Yeh, Chi-Hsuan Wei, Wann-Neng Jane, Mei-Chu Chung, Co-Shine Wang

**Affiliations:** ^1^Graduate Institute of Biotechnology, National Chung Hsing University, Taichung 40227, Taiwan; ^2^Institute of Plant and Microbial Biology, Academia Sinica, Nankang, Taipei 11529, Taiwan

**Keywords:** Adhesin, anther, exine, hormone, lily (*Lilium longiflorum* Thunb.), microspore, tapetum.

## Abstract

The anther-specific gene *LLA1271* isolated from lily (*Lilium longiflorum* Thunb.) anthers is novel and exists in two forms. The protein encoded by *LLA1271* may represent an adhesin-like protein first found in higher plants. The protein contains a typical N-terminal signal peptide followed by a highly conserved repeat domain. The *LLA1271* gene is temporally expressed at the phase of microspore development. RNA blot and RNA *in situ* hybridization analyses demonstrated that the gene was expressed both in the tapetum and in the microspore. The gene is endo- and exogenously induced by gibberellin. Studies with the gibberellin biosynthesis inhibitor uniconazole and an inhibitor of ethylene activity, 2,5-norbornadien (NBD), revealed that *LLA1271* is negatively regulated by ethylene, and a cross-talk of regulation between gibberellin and ethylene occurs in young anthers. The treatment with NBD caused the tapetum to become densely cytoplasmic and highly polarized, whereas uniconazole arrested tapetal development in a state close to that of a tapetum without treatment. The LLA1271 protein is heat stable and heterogeneous. An immunoblot of separated protein fractions of the anther revealed that the LLA1271 protein was detected in protein fraction of the microspore released from the cell wall by treatment with either 0.5% or 2% Triton X-100. Ectopic expression of *LLA1271* resulted in impaired stamen and low pollen germination. Scanning electron microscopy of *TAP::LLA1271* pollen showed distorted exine formation and patterning. The LLA1271 protein once synthesized in both the tapetum and microspore is secreted and deposited on the surface of microspores, moderately affecting exine formation and patterning.

## Summary

The lily anther-specific gene, *LLA1271*, encodes an adhesin-like protein found for the first time in higher plants. The protein is deposited on the surface of microspores, moderately affecting exine formation.

## Introduction

In higher plants, pollen formation occurs in a specialized floral organ, the stamen. The young anther is composed primarily of sporogenous tissue surrounded by a number of wall layers. Of these, the tapetum, the innermost layer of the anther wall, represents a secretory tissue providing nutrition and other resources necessary for the developing microspores (Wilson and Zhang, 2009). The tapetum synthesizes and secretes various proteins, enzymes, sporopollenin precursors, lipidic molecules, and many other compounds into the anther loculi and finally in cavities between the pollen exine, completing the pollen coat and exine structure (Liu and Fan, 2013). Without a differentiated tapetum, critical nutrient and required resources are not available for proper microspore/pollen development (Wilson and Zhang, 2009). The intimate interaction between tapetum and microspore/pollen ensures the fertility of pollen grains and the interaction is modulated by a sophisticated network of hormone interplay (Plackett *et al.*, 2011). Gibberellin (GA) has been found to be synthesized in the tapetum (Kaneko *et al.*, 2003). A few tapetal genes have previously been reported to be regulated by GA in anthers (van den Heuvel *et al.*, 2002; Tzeng *et al.*, 2009). However, knowledge of gene regulation specifically involved in the early programme of male gametogenesis is rather limited.

Concomitant with differentiated tapetum, the wall layers of microspores derived from sporogenous cells become specialized. A temporary cell wall (callose) encompasses each microspore and later is removed enzymatically (Enns *et al.*, 2005). The layer of primexine, a precursor to the ectexine, is then deposited at the microspore surface, followed by the endexine and finally the intine. The subsequent addition of tapetally derived sporopollenin and other molecules drastically alters the structure of the ectexine where the elements such as the tectum and columella are developed to become less elastic during the free microspore stage (Blackmore *et al.*, 2007). In addition to layering, pollen wall patterning also involves the development of germinal apertures on the wall of microspores. A limited number of studies on the stamen have dealt with pollen wall development. There are several *Arabidopsis* mutants defective in exine formation, and these have been characterized in detail. These mutated proteins may be enzymes involved in the formation of sporopollenin and fatty acid metabolism (Yi *et al.*, 2010; Chen *et al.*, 2011; Colpitts *et al.*, 2011). Some of them are transporters involved in the transport of substrates such as sporopollenin monomers from the tapetum to microspores (Choi *et al.*, 2011; Qin *et al.*, 2013) and others are wall-associated proteins involved in the assembly and maintenance of the primexine and membrane (Zhang *et al.*, 2010; Sun *et al.*, 2013).

In order to identify specific genes in young anthers, a subtractive cDNA library at the stage of microspore development was constructed (Hsu *et al.*, 2008). Many genes specific to the stage of microspore development have been identified, among which *LLA1271* (*Lilium longiflorum anther 1271*) was analysed further. Here, the gene expression and regulation of *LLA1271* are characterized and the physiological role of its product is proposed. The gene is controlled by a cross-talk between GA and ethylene in young anthers. The proteins encoded by *LLA1271* may represent novel cell surface adhesin-like proteins in lily anthers. The adhesin-like proteins have non-covalent binding strength with the exine wall. Scanning electron microscopy (SEM) of the *TAP::LLA1271* pollen reveals distorted exine formation and patterning. Thus, the LLA1271 proteins may be associated with the early stage of exine development.

## Materials and methods

### Plant material and treatments

Plants of lily (*Lilium longiflorum* Thunb. cv. Snow Queen) were grown in the field. Buds of various sizes were dissected to isolate anthers according to Tzeng *et al.* (2009). Meiosis occurred in the pollen mother cells at a bud size of ~20–25mm, resulting in the formation of tetrads. After microspore mitosis was complete at a bud size of ~65–70mm, pollen subsequently entered the maturation phase of development. Concomitant with the development of the microspore, the tapetum of the anther wall became secretory and then degenerate afterwards. The addition of GA_3_, 100 μM uniconazole, or 100 μM 2,5-norbornadien (NBD) was also described in Tzeng *et al.* (2009). For the treatment with inhibitors, the cut plants with 17–20mm buds continued to grow to 21–24mm in bud size (~4 d) before decapitation.

### Plasmid isolation, PCR, and sequence analysis

The *LLA1271* cDNA clone was identified from a subtractive cDNA library at the stage of microspore development in lily anthers (Hsu *et al.*, 2008). Plasmid DNA was purified and the cDNA cloned in the pGEM-T Easy vector (Promega, Madison, WI, USA) was digested with *Rsa*I to determine the insert size. 5′- and 3′-rapid amplification of cDNA ends (RACE) PCR was carried out according to the user manual of the SMART™ RACE cDNA amplification kit (Clontech Laboratories, Inc., Mountain View, CA, USA). For the identification of two forms of *LLA1271*, 5′-RACE PCR was performed with primers A (5′-CAGAAGATAGAAAAACAGTAACCACGGC-3′) and B (5′-CAGCCATTATTCCACCCCAAGCTACTGC-3′), respectively. The PCR products were fractioned using a 1.5% agarose gel and stained with ethidium bromide. For reverse transcription–PCR (RT–PCR) analysis, total RNA was isolated from 5-week-old inflorescence of the wild type and the two *TAP::LLA1271* transgenic lines 11 and 25. The fragment of *LLA1271* was amplified using a pair of primers (5′-CGCGGATCCATGGCGAAACTCAGCTTCTG-3′) and (5′-CGAGCTCTCAAACTCCAACTTTTAAGGG-3′). DNA sequence from both strands of the cloned inserts was obtained using an ABI model 377 (Foster City, CA, USA) automated sequencer. Sequence alignment was achieved using the Vector NTI Suite 8 program (InforMax, Inc., Bethesda, MD, USA) and the homology search was done with the BLAST program (Altschul *et al.*, 1997).

### RNA blot

The method of separating microspores from the anther wall described below is similar to the method described by Aouali *et al.* (2001). Anthers of young buds >34mm were sliced open transversely with a scalpel. Microspores were gently squeezed out into a buffer of 10mM sodium acetate, pH 5.2. After centrifugation at 5000 *g* for 3min, the pellet (microspores) was ready for the extraction of total RNA. Total RNA was extracted from developing anthers and from other floral and vegetative organs using the Ultraspec RNA isolation system (Biotecx Laboratories Inc.). Total RNA samples were electrophoresed in 1.0% formaldehyde–MOPS gels and transferred onto nylon membranes (Micron Separation Inc.), after which membrane pre-hybridization and hybridization were carried out according to standard procedures (Sambrook *et al.* 1989).

### RNA *in situ* hybridization

The method was as described by Tzeng *et al.* (2009). Digoxigenin (DIG)-labelled RNA probes were synthesized using a DIG RNA labelling kit (SP6/T7) (Roche Diagnostics GmbH, Penzberg, Germany). The hybridization signal viewed under a bright-field microscope is brownish purple. Sections were counterstained with 0.001% Fast Green.

### Preparation, electrophoresis, and immunoblotting of lily protein

The phenol extraction method was used to extract protein from various vegetative and floral organs of lily plants (Wang *et al.*, 1992). Total protein extracted from anthers of 34–46mm buds was subjected to two-dimensional polyacrylamide gel electrophoresis (2D-PAGE) and either stained with Coomassie blue or electroblotted onto nitrocellulose (0.45 μm, Millipore Corporation, Billerica MA, USA). The extraction of heat-stable proteins from anthers of 34–46mm buds was performed according to Wang *et al.* (1996). The heat-soluble proteins and resolubilized pellet were fractionated by SDS–PAGE and either stained with silver or Coomassie blue or electroblotted onto nitrocellulose. Antiserum was raised against a synthetic peptide SAKLHAVSESVKPSAK designed from a segment of LLA1271 (Supplementary Fig. S1 available at *JXB* online), which is conjugated with the carrier, keyhole limpet haemocyanin. Antibodies were affinity-purified using the method of Smith and Fisher (1984) and used for immunoblot analysis.

### Preparations of fractions of the anther wall, the exine-released protein fraction, and the microspore after protein release treatment

Anthers of 34–46mm buds were sliced open transversely with a scalpel. Microspores were gently squeezed out into a buffer of 10mM sodium acetate, pH 5.2. After centrifugation at 5000 *g* for 3min, the pellet (microspores) was placed in a new microcentrifuge tube containing 1ml of 50mM sodium acetate, pH 5.2 with or without either 0.5% or 2% Triton X-100, shaken gently for 20min, and centrifuged. The supernatant was collected as the exine-released protein fraction. Protein was extracted from each fraction by the phenol extraction method as described previously (Wang *et al.*, 1992).

### Constructs and *Arabidopsis* transformation

To generate the construct, the *LLA1271a* cDNA was amplified by PCR using *pLLA1271a* cDNA as a template with a 5′-primer (5′-CGCGGATCCATGGCGAAACTCAGCTTCTG-3′) and 3′-primer (5′-CGAGCTCTCAAACTCCAACTTTTAAGGG-3′) pair. The resulting PCR fragment of *LLA1271a* was digested with *Bam*HI and *Sac*I, and subcloned into the pBI101 vector (BD Biosciences Clontech) that was also digested with *Bam*HI and *Sac*I. Then, 1.2kb of the *RTS* gene regulatory region (*TAP*) (Luo *et al.*, 2006) was digested with *Bam*HI and subcloned into the pBI101 vector. *TAP* is a kind gift from Dr H. Luo, Department of Genetics, Clemson University. The coding sequence of *LLA1271a* and *TAP* in the construct was verified before subcloning. Transformation of *Arabidopsis* was according to the vacuum infiltration method (Bechtold *et al.*, 1993) using *Agrobacterium tumefaciens* strain LBA4404. Seeds were harvested and plated on kanamycin selection medium (50 μg ml^–1^) to identify T_1_ transgenic plants. T_2_ progeny homozygous for kanamycin resistance were used for further studies.

### Pollen germination

Germination was examined according to the method of Rodriguez-Enriquez *et al.* (2013) with some modifications. The germination medium consisted of all components with the exception of 0.03% casein enzymatic hydrolysate, and was adjusted to pH 8.0. To avoid heat damage, 0.1mM spermidine (Sigma) and 10mM GABA (Sigma) were added to the medium only after 0.5% agarose had completely heat-dissolved in a microwave and cooled to 42 °C. Pollen was layered on the surface of the cellophane membrane-covered agarose pad placed on an uncoated glass slide and vertically incubated in the dark at 22 °C for 16h before examination.

### Phenotype characterization and microscopy

Flower images were taken using an Olympus dissection microscope with an Olympus digital camera. Alexander solution and staining were performed as described (Alexander, 1969). Photography was performed with an Olympus SZX7 microscope. Transverse sections of anthers of 17–20mm buds treated or not with 100mM uniconazole and/or 100mM NBD for 4 d. Sections were stained with 1% safranine O in 50% ethanol. Mature flowers of *Arabidopsis* were fixed in 2.5% glutaraldehyde and 4% paraformaldehyde in 0.1M sodium phosphate buffer, pH 7.0 at room temperature for 4h. After three 20min buffer rinses, the samples were post-fixed in 1% OsO_4_ in the same buffer for 4h at room temperature and then rinsed in three 20min changes of buffer. Samples were dehydrated in an ethanol series and propylene oxide, embedded in Spurr’s resin, and sectioned with a Lecia Reichert Ultracut S or Lecia EM UC6 ultramicrotome. The ultra-thin sections (70–90nm) were stained with uranyl acetate and lead citrate. Sections were observed using a Philips CM 100 transmission electron microscope at 80kV and the images were obtained with a Gatan Orius CCD camera. For SEM, pollen grains were coated with platinum particles (JFC-1600) for 30 s at 20 mA and viewed under a JSM-7401F microscope (JEOL).

## Results

### Cloning and sequence analysis of *LLA1271a* and *LLA1271b* cDNAs

**Fig. 1.**
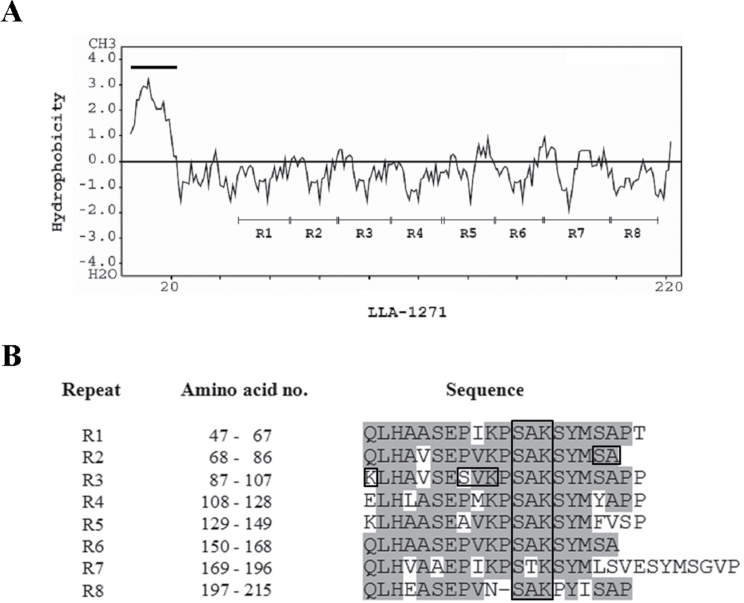
Hydropathy profile and alignment of eight repeat sequences of LLA1271a. (A) Hydropathy profiles of LLA1271a protein sequence. The black line indicates a hydrophobic sequence at the N-terminus of the sequence. The eight repeat sequences are indicated as R1–R8. (B) Sequence alignment of eight repeats. The box indicates the putative phosphorylation (S/T-X-K/R) sites.

**Fig. 2.**
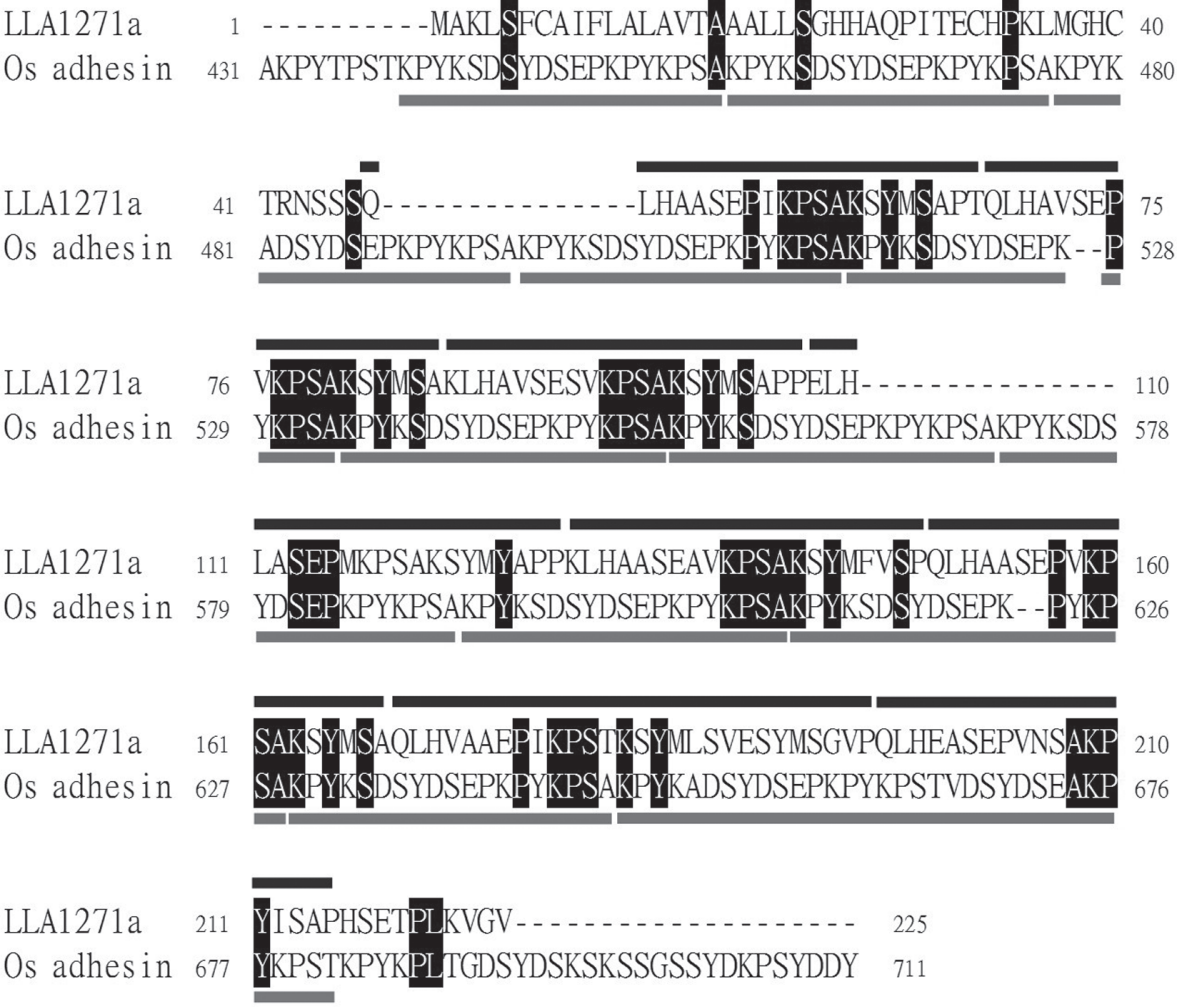
Alignment of lily LLA1271a with Os adhesin, a GLEYA adhesin domain protein. The deduced amino acid sequence of LLA1271a from *Lilium longiflorum* (accession no. EF026009) is aligned with a GLEYA adhesin domain protein (Os adhesin, accession no. EQL00008.1) of *Ophiocordyceps sinesis* C018 at the C-terminus. The amino acid residues that are identical between the two sequences are highlighted by black blocks. The black and grey lines indicate the conserved tandem repeats in LLA1271a and Os adhesin, respectively.

The *LLA1271a* clone obtained from a subtractive cDNA library (Hsu *et al.*, 2008) contains only a partial insert; 5′- and 3′-RACE PCR was used to obtain the full-length *LLA1271a* cDNA. It turns out that the *LLA1271* cDNA exists in two forms, as shown in Supplementary Fig. S1 available at *JXB* online. The sequence of *LLA1271a* (accession no. EF026009) is almost identical to that of *LLA1271b* (accession no. EU374582), except that a codon AGA in the coding region of *LLA1271a* is exchanged for the codons ACAGGA in *LLA1271b* and a 76bp fragment in the 3′-untranslated region of *LLA1271a* is absent from *LLA1271b*. The two forms of *LLA1271* mRNAs were confirmed by RT–PCR. When the 3′-primer A was designed in the region of *LLA1271a* which in absent in *LLA1271b*, only a single PCR product was observed; with the 3′-primer B, two distinct DNA products were observed in the gel (Supplementary Fig. S2 available at *JXB* online). The *LLA1271a* cDNA encodes a polypeptide of 225 amino acids whereas that of *LLA1271b* encodes a polypeptide of 226 amino acids. The deduced amino acid sequence of *LLA1271* has a calculated molecular mass of ~24kDa. The hydropathy profile (Kyte and Doolittle, 1982) of LLA1271a shows that the polypeptide has an overwhelming hydrophilicity and allows a clear visualization of the repeats (Fig. 1A). The protein is hydrophilic with the exception of a strong hydrophobic region at the N-terminus, indicating the presence of a signal peptide (Fig. 1A; Supplementary Fig. S1 available at *JXB* online). It is intriguing that the deduced LLA1271a and LLA1271b proteins contain eight highly conserved sequence repeats (Fig. 1B). In addition, the protein contains 10 putative phosphorylation sites (S/T-X-K/R) and one putative *N*-glycosylation (N-X-S/T) site. The predicted amino acid sequence of *LLA1271a* was utilized to search protein databases. Sequence alignment analysis revealed significant similarity between the predicted LLA1271a polypeptide and a recently identified GLEYA adhesin domain protein (Os adhesin, accession no. EQL00008.1) of *Ophiocordyceps sinesis* C018 at the C-terminus (Fig. 2). The Os adhesin contains 12 conserved repeats at the C-terminus whereas the LLA1271 (both LLA1271a and LLA1271b) proteins possess a domain of eight repeats (Fig. 2). The GLEYA adhesin domain proteins have only been found in fungi and fission yeasts to date (Linder and Gustafsson, 2008). Thus, *LLA1271* is likely to be the first adhesin-like gene found in higher plants.

### Organ specificity and temporal expression of the *LLA1271* gene

**Fig. 3.**
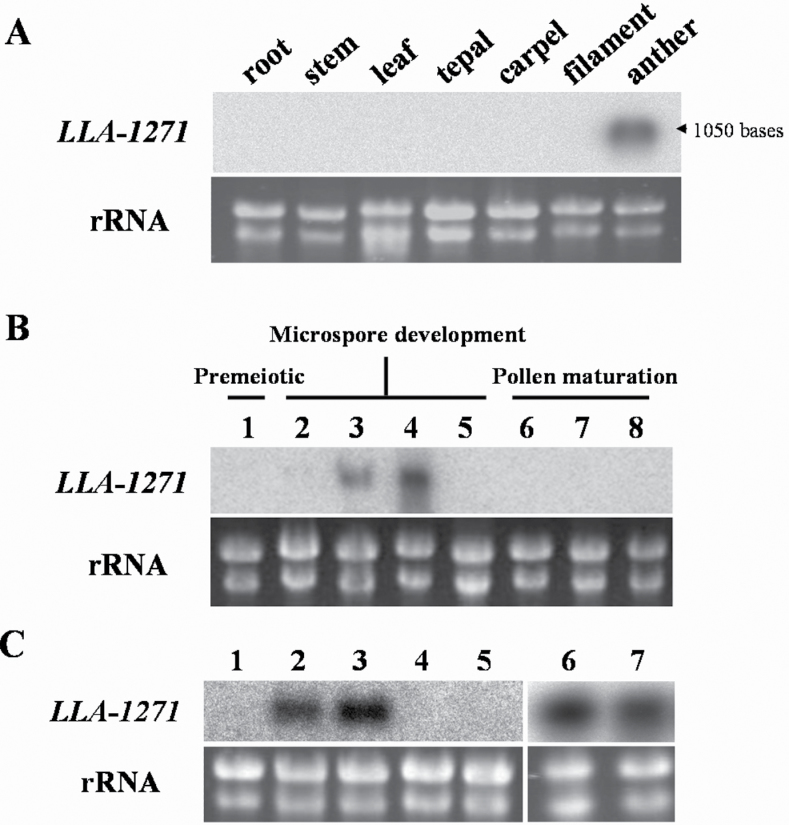
Organ specificity and temporal expression of *LLA1271* in the anther of *L. longiflorum*. (A) Total RNA (20 μg) was isolated from various vegetative organs and floral organs of 34–46mm buds. (B) Total RNA (20 μg) was isolated from stamen/anthers of different sizes of buds: 1, <15mm buds; 2, 24–26mm buds; 3, 34–36mm buds; 4, 44–46mm buds; 5, 60–65mm buds; 6, 90–95mm buds; 7,120–125mm buds; 8,150–155mm buds. (C) Total RNA (20 μg) was isolated from anthers of 24–26mm buds (1) and from microspores of various sizes of buds: 2, 34–36mm buds; 3, 44–46mm buds; 4, 60–65mm buds; 5, 70–75mm buds; and from anthers (6) and anther wall (7) of 44–46mm buds. Total RNA was denatured, fractionated on formaldehyde–agarose gels, transferred to nylon membranes, and hybridized to the ^32^P-labelled *LLA1271a* cDNA insert. Almost equal amounts of total RNA were loaded in each lane, as determined by ethidium bromide staining of the gel.

Total RNA was isolated from vegetative organs (root, stem, and leaf) and floral organs in 34–46mm buds (tepal, filament, anther, and carpel, comprising the stigma, style, and ovary) to determine the organ specificity of *LLA1271* gene expression in lily plants. The isolated mRNA on a blot was hybridized with ^32^P-labelled *LLA1271a* cDNA (Fig. 3A). Hybridization signals were only detected in the RNA samples from the anther, indicating that the gene was organ specific.

**Fig. 4.**
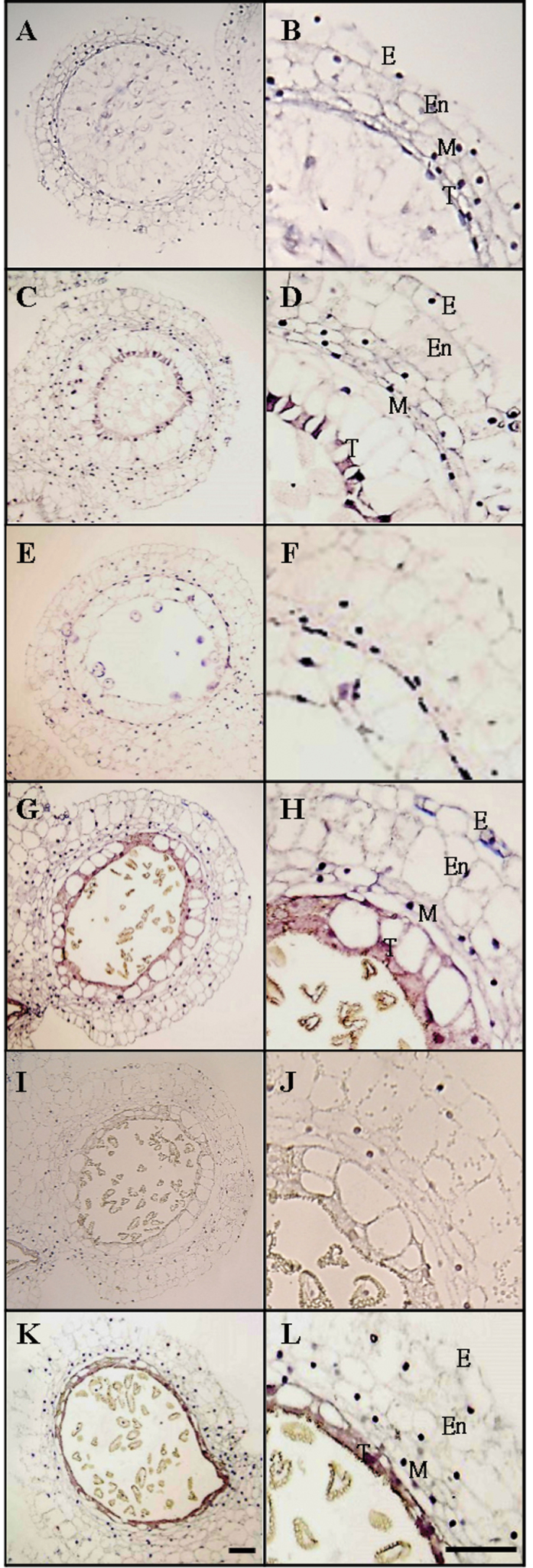
*In situ* hybridization of *LLA1271* transcripts in the developing anther of *L. longiflorum.* Expression of *LLA1271* was analysed in bright-field illuminated 7–10 μm cross-sections of anthers of 20–25mm (A and B), 35–40mm (C-F), 45–50mm (G-J), and 55–60mm (K and L) buds using DIG-labelled sense (E, F, I, and J) and antisense (A–D, G, H, K, and L) *LLA1271a* RNA probes. E, epidermis; En, endothecium; M, middle layer; T, tapetum. Bar=100 μm.

Blots of total RNA isolated from anthers of lily buds of varying size classes were hybridized with ^32^P-labelled *LLA1271a* cDNA to determine the expression pattern of *LLA1271* during anther development. The *LLA1271* transcript was first detected in anthers of 34–36mm buds. The gene accumulated its mRNA to a maximum level around anthers of 44–46mm buds. No hybridization signal of *LLA1271* was detected in the anther of 60–65mm buds (Fig. 3B). Signals were not detected before meiosis and during pollen maturation. The changing pattern of *LLA1271* gene expression corresponds to microspore development and major cytological changes in the wall layers (Fig. 4).

Anthers of different size classes were dissected into two parts, the anther wall and the microspores, to determine further whether the gene was expressed in the microspores. A blot of total RNA isolated from microspores at various developmental stages was hybridized with ^32^P-labelled *LLA1271a* cDNA. In addition to those characterized in the anther wall (tapetum), *LLA1271* transcripts were coordinately detected in the various stages of microspores in buds of 34–46mm (Fig. 3C). No signals were detected from blots of mRNA isolated from the microspores/pollen in 60–65mm buds or thereafter (lanes 4 and 5), reinforcing the stage specificity of *LLA1271* gene expression in the anther/microspores. Signal detected in the anther or anther wall (lanes 6 and 7) was used as a positive control.

### *In situ* localization of *LLA1271* mRNAs in developing lily anthers

*In situ* hybridization with the DIG-labelled antisense RNA probe of the *LLA1271a* gene was performed to determine the cellular location of the transcripts. No signal was visible in sections of anthers in 35–40mm buds that were treated with sense probes (Fig. 4B). When the DIG-labelled antisense riboprobe of *LLA1271* was used, hybridization signals with dark brownish purple signals were observed in the tapetum of 35–40mm buds (Fig. 4C, D), whereas no signal was visible in sections of anthers in 20–25mm buds (Fig. 4A). At this time, the tapetal cells became polarized and highly secretory in the anther. The brownish purple signals observed in the tapetum indicated that the transcripts of the *LLA1271* gene were significantly expressed in the tapetum compared with the other anther wall layers. However, hybridization signals in the microspores were not discernible due to their distorted structure. As buds grew to 45–50mm, the tapetal cells began to disintegrate in the anther (Fig. 4E, F) and the strength of hybridization signals decreased compared with the cells observed in the tapetum of 35–40mm buds (Fig. 4G, H).

### GA induces the expression of *LLA1271*

**Fig. 5.**
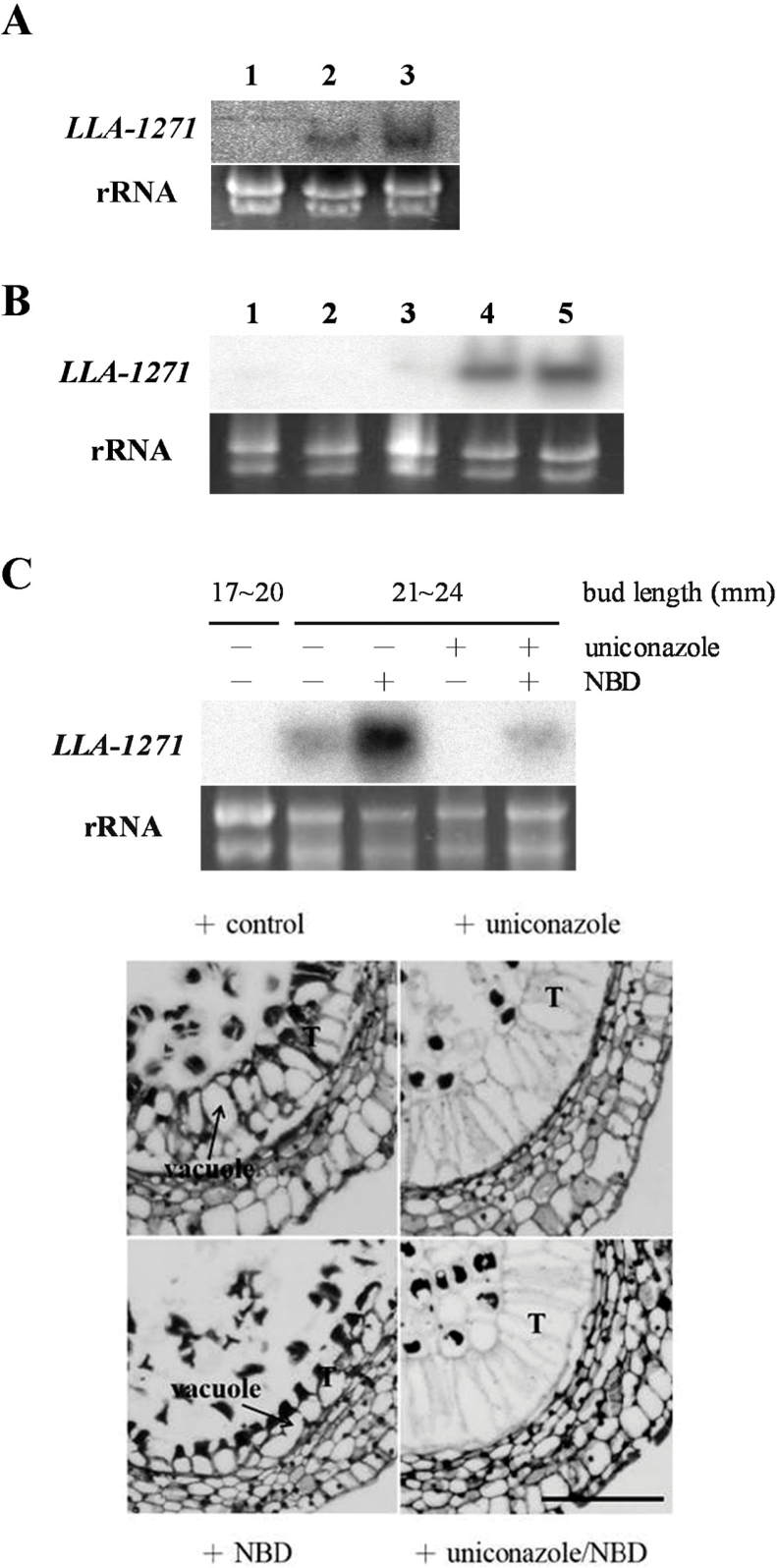
Expression and regulation of *LLA1271* by GA and ethylene in young anthers of *L. longiflorum*. (A) The 18–22mm buds dissected from lily plants were dipped in solutions without GA_3_ (1) or containing 0.1 μM (2) or 1.0 μM GA_3_ (3) for 24h. (B) The 24–26mm buds dissected from lily plants were dipped in a solution containing 1 μM GA_3_ for (1) 0h, (2) 3h, (3) 8h, (4) 24h, and (5) 36h. (C) The 17–20mm buds dissected from lily plants were dipped in a solution with or without treatment with 100 μM uniconazole and/or 100 μM NBD for 4 d, after which the bud size grew to 21–24mm. Total RNA (20 μg) was isolated from anthers, denatured, fractionated on formaldehyde–agarose gels, transferred to nylon membranes, and hybridized to the ^32^P-labelled *LLA1271a* cDNA insert (upper panel). Almost equal amounts of total RNA were loaded in each lane, as determined by ethidium bromide staining of the gel. The enlarged transverse sections (lower panel) of anthers around 20mm buds with or without treatment were stained with 1% safranine O in 50% ethanol. T, tapetum. Bar=200 μm.

GA was exogenously applied to investigate its inducing effect on gene expression. Cut lily plants with 18–22mm buds were dipped in solutions containing various concentrations of GA_3_ for 24h, after which total RNA was extracted from the anther. RNA blot analysis revealed that the *LLA1271* mRNA enhanced its level of accumulation even when as little as 0.1 μM GA_3_ was applied (Fig. 5A).

In order to determine the kinetics of the GA_3_-stimulated accumulation of *LLA1271* mRNAs in lily anthers, a time-course experiment was conducted. The young lily plants with 24–26mm buds were dipped in a solution containing 1 μM GA_3_ for various times. RNA blot analysis showed that a significant level of *LLA1271* mRNA accumulation occurred at 8h of GA_3_ treatment and thereafter (Fig. 5B).

### A cross-talk between ethylene and GA regulates gene expression of *LLA1271*

GAs are produced in the tapetum (Kaneko *et al.*, 2003). To examine further whether the *LLA1271* gene is stimulated by GA and other hormones (endogenous) in the anther, uniconazole, a potent inhibitor of GA biosynthesis, and NBD, an effective inhibitor of ethylene action, were applied. Plants with 17–20mm buds were used instead because no signal of *LLA1271* mRNA was detected in the anther at this period. Plants with 17–20mm buds were dipped in a solution with or without 100 μM uniconazole and/or 100 μM NBD and continuously grown to 21–24mm (~4 d), after which total RNA was extracted from the anther. The treatment with uniconazole did not cause any visible growth retardation of lily buds, whereas NBD extended the bud length to a size larger than 28mm after a 4 d treatment. RNA blot analysis revealed that without the addition of both inhibitors, the *LLA1271* mRNA accumulated in the anther after buds grew from 17–20mm to 21–24mm (Fig. 5C, upper panel). However, no signal of mRNA was detected when the same size of lily buds (24–26mm) was analysed in Figs 5A and 3B. The inconsistency is because lily plants grown in the field were harvested at different seasons, resulting in a slight shift of the gene expression pattern to earlier or later during development. With the treatment with 100 μM uniconazole, the accumulation of mRNAs was completely inhibited, indicating that the gene is induced by endogenous GA. Further, the treatment with uniconazole arrested tapetal development at a state similar to that of the control (Fig. 5C, lower panel). In contrast, the accumulation of *LLA1271* mRNA was intensely enhanced if 100 μM NBD was applied (Fig. 5C, upper panel). The treatment with NBD caused the tapetum in the anther to become densely cytoplasmic and highly polarized such that the vacuole was towards the outside and the cytoplasm towards the locular side (Fig. 5C, lower panel). It is obvious that growth of the tapetal cells was more advanced than that of the control. This clearly indicates that the gene is negatively regulated by ethylene. When plants were treated with both inhibitors, the *LLA1271* mRNA prominently decreased its level of accumulation when compared with plants only treated with NBD, but the tapetal morphology was similar to that of plants treated with uniconazole only (Fig. 5C, lower panel). These findings reinforce the concept that GA is the main hormone regulating *LLA1271* expression, and ethylene negatively modulates GA-induced *LLA1271* gene expression.

### The anther-specific LLA1271 protein is heterogeneous, heat stable, and accumulates only in the phase of microspore development

**Fig. 6.**
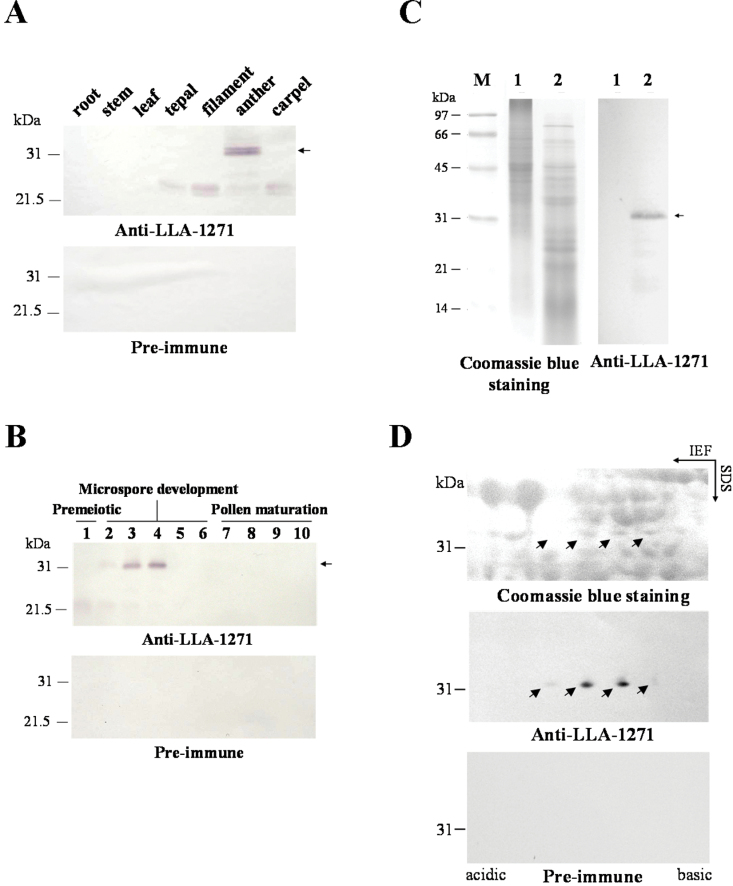
Immunological characterization of the LLA1271 protein in the anther of *L. longiflorum.* (A) Total protein (40 μg) was isolated from various vegetative organs of lily pants and floral organs of 34–46mm buds. (B) Total protein (40 μg) was isolated from stamen/anthers of different sizes of buds: 1, 10–20mm buds; 2, 20–30mm buds; 3, 30–40mm buds; 4, 40–50mm buds; 5, 50–60mm buds; 6, 70–80mm buds; 7, 90–100mm buds; 8, 110–120mm buds; 9, 130–140mm buds; 10, 150–160mm buds. (C) Total protein was isolated from anthers of 34–46mm buds and heat treated at 90 ºC for 10min. The resolubilized pellet (precipitation, lane 1) and heat-soluble protein (supernatant, lane 2) were fractionated by SDS–PAGE. (D) Total protein (1.2mg) extracted from anthers of 34–46mm buds was electrophoresed by 2D-PAGE. The gels were either stained with Coomassie blue or electroblotted onto nitrocellulose and immunologically detected using affinity-purified LLA1271 or pre-immune antibodies at a 1:10 000 dilution. The LLA1271 polypeptides are indicated by arrows. Marker proteins are indicated on the left.

Total protein was extracted from both vegetative and floral organs of lily plants and from various size classes of anthers. Protein was then fractionated by SDS–PAGE to determine organ specificity and developmental accumulation of the LLA1271 protein. Immunoblots of total protein revealed that a 32kDa protein was recognized by affinity-purified LLA1271 antibodies only in the anther (Fig. 6A) and only at the microspore stage during anther development (Fig. 6B). A weak band of 22kDa protein was also detected in other floral organs of lily plants and at the pre-meiotic phase of anther development. No protein was detected when identical blots were incubated with affinity-purified antibodies from pre-immune serum. The organ specificity and temporal distribution of LLA1271 during development are consistent with previous results analysed by RNA blots (Fig. 3).

The heat stability of LLA1271 was examined by heating at 90 °C for 10min. After centrifugation, the heat-soluble fraction and the pellet (precipitation) were subjected to SDS–PAGE (Fig. 6C). An immunoblot revealed that a 32kDa polypeptide was recognized by affinity-purified LLA1271 antibodies in the heat-soluble fraction, suggesting that the LLA1271 protein is heat stable (Fig. 6C). To determine the heterogeneity of LLA1271, total protein extracted from anthers of 34–46mm buds was fractionated by 2D-PAGE. An immunoblot of total protein revealed that an array of two major and two minor polypeptides (indicated by arrows) that differed in isoelectric point was recognized by affinity-purified LLA1271 antibodies, while no protein was recognized by pre-immune antibodies (Fig. 6D). This indicates that the protein is heterogeneous.

### Fractionation of distinct structures or origins from developing anthers of *L. longiflorum*

**Fig. 7.**
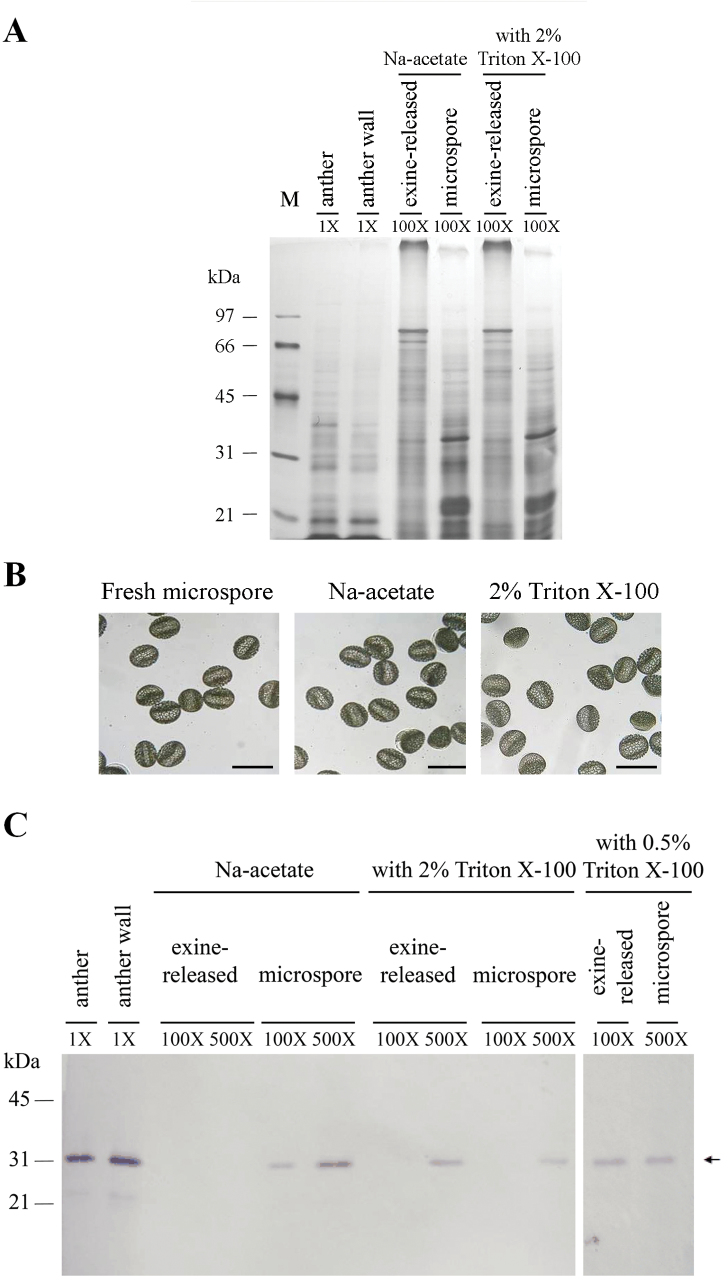
Distribution of the LLA1271 protein in fractions of distinct origins separated from anthers of *L. longiflorum*. SDS–PAGE of proteins in the anther of 34–46mm buds (total) and separated fractions including the anther wall, exine-released, and microspore fractions. Proteins were released from the exine layer of microspores by an aqueous solution of sodium acetate with or without the addition of either 0.5% or 2% Triton X-100. The gel was either stained with silver (A) or electroblotted onto nitrocellulose and immunologically detected using affinity-purified LLA1271 antibodies at a 1:20 dilution (C). Molecular mass markers in kDa are indicated on the left side. Different proportions of individual samples were applied to the lanes, and these proportions relative to an equal amount of the anther are shown in the gel. (B) Light microscopic photographs of fresh microspores, and microspores after treatment with an aqueous solution with or without 2% Triton X-100. The scale bar represents 100 μm.

To investigate the distribution of LLA1271 proteins in the anther, anthers of 34–46mm buds were separated into three distinct fractions: the anther wall, a protein fraction released from the wall layer (exine) of microspores, and the microspore produced by the protein release treatment. These fractions were analysed for their protein constituents by SDS–PAGE. The anther wall, exine-released, and microspore proteins resolved in the gel were basically non-overlapping, which indicates the selectivity of the separation procedure (Fig. 7A). The microspores remained intact under the treatment with 2% Triton X-100 when examined by microscopy (Fig. 7B). An immunoblot of proteins from each fraction revealed that a 32kDa polypeptide was recognized by affinity-purified LLA1271 antibodies in the exine-released protein fraction if microspores were treated with 2% Triton X-100 while no protein was recognized if treated without the addition of Triton X-100 (Fig. 7C). The LLA1271 protein was released in the exine-released protein fraction even with the treatment by 0.5% Triton X-100. In parallel with the exine-released protein fraction, the LLA1271 protein retained in the microspore adversely decreased its level with the treatment by 2% Triton X-100 when compared with that without the addition of Triton X-100, suggesting that the protein has non-covalent binding strength with the exine structure. The detection of LLA1271 protein in the anther and the anther wall was used as a positive control.

### Ectopic expression of *LLA1271* results in impaired stamen and distorted exine patterning of pollen grains

**Fig. 8.**
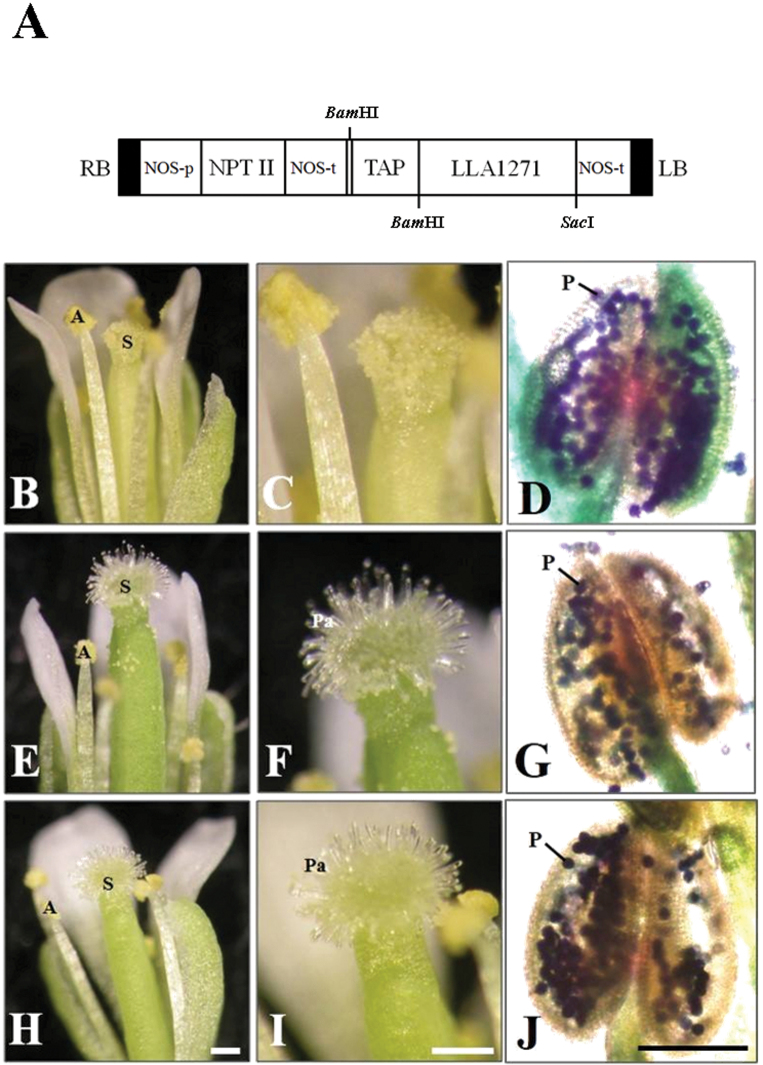
Generation and phenotype analysis of *TAP::LLA1271* transgenic lines. (A) Construct of *LLA1271* fused with the *RTS* gene regulatory region (*TAP*) for plant transformation. RB, right T-DNA border; LB, left T-DNA border; NPTII, neomycin phosphotransferase II; NOS-p, NOS promoter; NOS-t, NOS terminator. Phenotypes of the wild type (B–D) and two *TAP::LLA1271* transgenic lines (E–J) were observed using a dissection microscope. Pollen grains of the wild type (D) and *TAP::LLA1271* transgenic lines (G, J) were stained with Alexander’s staining for viability testing. A, anther; S, stigma; Pa, papillae; P, pollen. Scale bars=200 μm (B, C, E, F, H, I) and 100 μm (D, G, J).

**Fig. 9.**
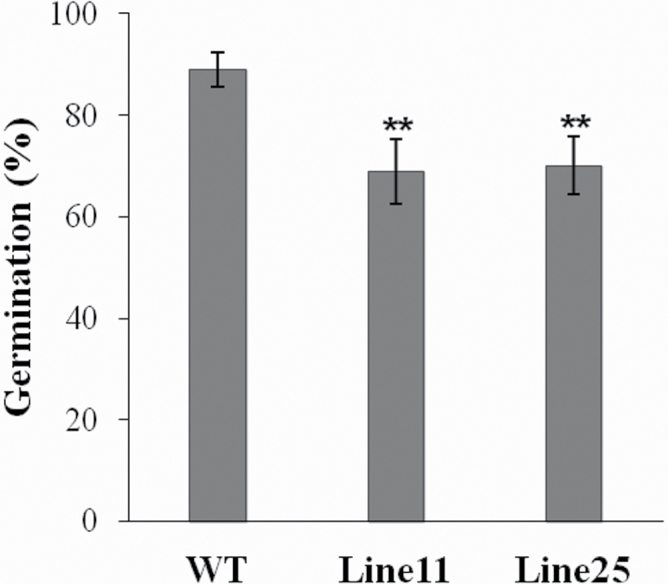
Germination of *TAP::LLA1271* pollen grains. Pollen of the wild type and two *TAP::LLA1271* transgenic lines was germinated *in vitro* in the germination buffer for 16h. At least 500 pollen grains were scored in duplicate for each germination test. The data were obtained from three independent experiments. Error bars represent the SD (*t*-test: **P<0.01).

To examine the function of LLA1271, an overexpression approach was used. The coding region of *LLA1271a* was fused to the rice tapetum-specific *RTS* gene regulatory region (*TAP*) (Luo *et al.*, 2006) and the construct (Fig. 8A) was used to transform *Arabidopsis* [ecotype Columbia (Col)] plants. The *RTS* gene promoter directs anther-specific gene expression in both monocotyledonous and dicotyledonous plants (Luo *et al.*, 2006). T_1_ and T_2_ kanamycin-resistant *Arabidopsis* lines were recovered. Of the three T_2_ homozygous lines, two with higher *LLA1271* expression levels were selected for more detailed analysis. RT–PCR analysis confirmed that the transcripts were present in the inflorescence of both transgenic plants harvested at 5 weeks, whereas no expression was detected in the wild type, as expected (Supplementary Fig. S3 available at *JXB* online). The growth and development of LLA1271-overexpressing plants in soil in the growth chamber appeared normal under normal growth conditions. Wild-type flowers had a large number of pollen grains on the stigmas (Fig. 8B, C), but in *TAP::LLA1271* plants, stamen development was impaired and few pollen grains were observed on the stigmas (Fig. 8E, F, H, I). Alexander’s staining (Alexander, 1969) was used to test the viability of pollen grains. Wild-type pollen grains were stained with purple colour (Fig. 8D), suggesting that they are viable. In contrast, it was observed that relatively high amounts of green-stained pollen remnants filled the anther in *TAP::LLA1271* (Fig. 8G, J), which indicated that these pollen grains were probably aborted. The viability of pollen grains was further examined by germination assay *in vitro*. It revealed that pollen of the two *TAP::LLA1271* lines exhibited a lower germination percentage (~70%) than that (90%) of the wild type (Fig. 9).

**Fig. 10.**
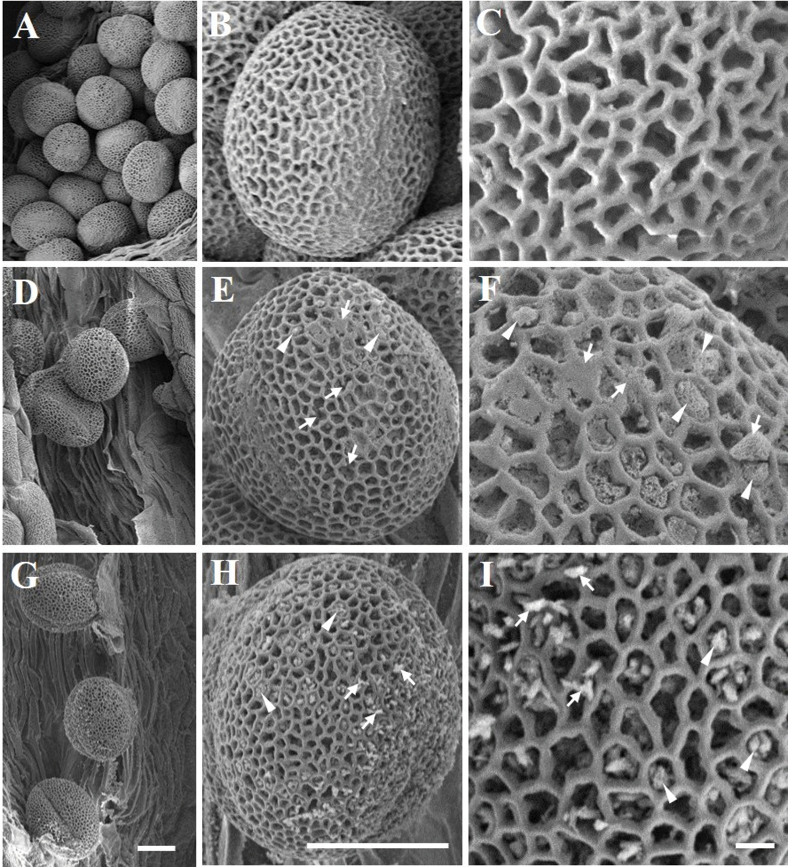
Scanning electron micrographs of *TAP::LLA1271* pollen grains. SEM micrographs of pollen grains from the wild type (A-C) and the two *TAP::LLA1271* transgenic lines 11 (D–F) and 25 (G–I). The two *TAP::LLA1271* pollen grains show defects in tectum formation. Two independent experiments were carried out, both with similar results. Arrowheads indicate limited areas without a visible exine network. Arrows indicate spherical or amorphous extrabacular protrusions in disconnected or smooth exine. Scale bars=10 μm (A, B, D, E, G, H) and 1 μm (C, F, I).

The size of *TAP::LLA1271* pollen was similar to that of the wild type, and SEM and transmission electron microscopy (TEM) were used to investigate exine formation and structure in the wild-type and *TAP::LLA1271* pollen. SEM revealed that wild-type pollen grains showed a typical round shape with reticulate exine (Fig. 10A–C). While a conserved exine pattern was present in the LLA1271-overexpressing pollen, the exine tecta appeared thinner or more distorted in *TAP::LLA1271* relative to the wild-type grains (Fig. 10D–I). Higher magnification images showed limited areas without a visible exine network (arrowheads). In addition, spherical or amorphous extrabacular protrusions and disconnected or smooth exine were observed in the *TAP::LLA1271* exine tecta (arrows in Fig. 10F, I). However, the exine structure and thickness of sporopollenin in the tecta were not discernible by TEM (Supplementary Fig. S4 available at *JXB* online). Taken together, these results suggest that LLA1271 is required for full exine wall integrity and patterning.

## Discussion

The *LLA1271* gene has been identified from a subtractive cDNA library at the stage of the microspore during anther development. The *LLA1271* gene is a novel anther-specific gene expressed both in the tapetum and in the microspore. Because the *LLA1271* gene exists in two forms whose sequences exhibit high similarity, the *LLA1271b* mRNA should also be detected using the radiolabelled *LLA1271a* probe in the analyses of RNA blots and RNA *in situ* hybridization. The spatial and temporal expression patterns of *LLA1271* are well correlated with tapetal development and degeneration. Around meiosis, when the buds are 20–25mm in length, the *LLA1271* mRNA and its protein do not accumulate in the anther. Accumulation levels reach a maximum when buds are ~40–50mm at which time the tapetum becomes highly secretory in the anther. Afterwards, expression of *LLA1271* significantly decreases as tapetal cells begin to disintegrate. The lily *LLA1271* gene is coordinately expressed in the tapetum and microspores during anther growth (Figs 3, 4). Apart from *LLA1271*, a number of lily genes co-expressed in tapetum and microspores have also been identified (Tzeng *et al.*, 2009; Liu *et al.*, 2011a, b). A chalcone synthase-like gene in tobacco and an anther-specific cysteine-rich protein in tomato also follow this expression pattern (Atanassov *et al.*, 1998; McNeil and Smith, 2005). Given that the LLA1271 protein is expressed both in the tapetum and in microspores, the specificity and application of a tapetum/microspore-specific promoter should warrant further investigation.

Tapetal cells in lily anthers are secretory and remain at the periphery of the microsporangium throughout development. In addition to LLA1271, the tapetum secretes various components into the locule. The secretory tapetum of *Lilium* also deposits flavonols, carotenoids, and lipids (termed pollenkitt) onto the pollen surface for normal exine formation (Reznickova and Dickinson, 1982). The growth of gametophytic microspores is profoundly dependent on the normal functionality of the sporophytic tapetum that secretes required resources into the anther loculi (Yang and Sundaresan, 2000; Scott *et al.*, 2004).

The tapetum is the source of GAs in the flower (Kaneko *et al.*, 2003). By adding 100 μM uniconazole to growing anthers, it was determined that GA indeed exists in young anthers and that the *LLA1271* gene is up-regulated by it. This result is consistent with earlier reports that have shown that GA is an endogenous plant growth regulator involved in the regulation of gene expression during anther development (van den Heuvel *et al.*, 2002; Tzeng *et al.*, 2009). Application of 100 μM NBD strongly increased the accumulation of *LLA1271* mRNA in the anther, indicating that the *LLA1271* gene is negatively regulated by ethylene (Fig. 5). The increased expression of *LLA1271* is correlated with the development of tapetal cells that become densely cytoplasmic and polarized. Thus, in addition to affecting anther gene expression, both uniconazole and NBD may seriously affect tapetal development. Upon treatment with both inhibitors, the *LLA1271* gene decreased its level of expression in comparison with the plants treated with only NBD. Thus, an antagonistic interaction between GA and ethylene is probably involved in the modulation of *LLA1271* gene expression in young anthers, although it is not clear at which level this cross-talk occurs. Few reports have demonstrated the involvement of GA and ethylene in tapetum/anther development (De Grauwe *et al.*, 2007; Hirano *et al.*, 2008). The antagonistic effect of ethylene on the GA activity occurs not only in the anther but also in other parts of floral organs. It was observed that after the treatment with NBD, lily buds grew larger than 25mm in comparison with other treatments where the standard size was 21–24mm. This observation coincides with a previous report (Kiss and Koning, 1989) where GA promoted cell elongation in both filament and corolla segments, while ethylene inhibited growth. Signal transduction pathways of various hormones form a complex and sophisticated network in plants (Steffens *et al.*, 2006). Multiple cross-talks may occur between GA and ethylene. Whether this cross-talk is additive, synergistic, or antagonistic is dependent on the process and on the individual stage of development in *Arabidopsis thaliana* (De Grauwe *et al.*, 2007).

It is striking that the *LLA1271* gene encodes a protein which contains eight highly conserved sequence repeats. Sequence alignment analysis reveals that the LLA1271 protein is similar to a GLEYA adhesin domain protein (Os adhesin) at the C-terminus (Fig. 2). Members of the family of GLEYA domain adhesins possess a typical N-terminal signal peptide and a domain of conserved sequence repeats, but lack glycosylphosphatidylinositol (GPI) anchor attachment signals (Linder and Gustafsson, 2008). Given that GLEYA adhesin domain proteins have only been found in fungi and fission yeasts to date (Linder and Gustafsson, 2008), *LLA1271* is probably the first adhesin-like gene found in higher plants.

The protein is rich in serine and proline within the repeats that are unfavourable for the protein to form β-sheets and α-helical structures. The lack of secondary structure in LLA1271 is reinforced by the fact that the protein is heat soluble when it is heat treated. Using 2D-PAGE, four isoforms of LLA1271 with different pIs were observed in the anther (Fig. 6D). Nevertheless, they are not easily discernible in the SDS gel analysis (Fig. 6C). The heterogeneity of LLA1271 may be due to changes in amino acid residues as supported by the fact that the *LLA1271* cDNA exists in two forms. In addition, they may arise by modifications of the primary translation products because the predicted amino acid sequence of *LLA1271* is 24kDa while the mature form is ~32kDa. The size differences between the predicted sequence and mature LLA1271 may be due to differences in the extent of modification based on the observation that the LLA1271 protein contains 10 putative phosphorylation sites (S/T-X-K/R) and one putative *N*-glycosylation site (N-X-S/T).

The LLA1271 protein has a hydrophobic N-terminal signal peptide. The presence of a putative signal peptide indicates the possibility that it is secreted since no retention signal (KDEL) (Verner and Schatz, 1988) is identified at the C-terminus of LLA1271. Further, the protein shares sequence similarity to Os adhesin, a member of the GLEYA domain adhesin proteins (Linder and Gustafsson, 2008). Thus, it is suggested that this signal peptide would target the growing polypeptide into the lumen of the endoplasmic reticulum, and the product is thereafter modified, and secreted from either the tapetal cells or the microspores into the wall layer (exine) of the microspore. The final deposition of the LLA1271 protein is confirmed by an immunoblot of the protein in the protein fraction released from the exine by treatment with Triton X-100 up to 2% (Fig. 7). This indicates that the LLA1271 proteins are bound non-covalently to the exine wall. However, it is not yet established how the protein is attached to the pollen wall.

Most fungal adhesins have a modular structure predominantly requiring C-terminal GPI anchors as a prerequisite for cell wall attachment. Once the adhesin is secreted into the membrane exterior, the GPI anchor is cleaved, followed by a covalent linkage to sugar moieties within the cell wall (Lu *et al.*, 1995). Apart from the GPI anchor attachment signals, there are alternative ways of attaching proteins to the cell wall in fungi. The WI-1/Bad1 adhesin only found in the Pezizomycotina is secreted into the external medium and subsequently connected to the cell wall by non-covalent binding to chitin chains through a process requiring tandem repeat sequences (Brandhorst and Klein, 2000). The LLA1271 polypeptide that contains a domain of eight highly conserved tandem repeats may possibly have the same binding function as the WI-1/Bad1 adhesin. An alternative example of attaching proteins to the cell wall without the GPI anchors is shown by a group of Pir proteins that become covalently linked to cell wall sugar molecules directly through glutamine residues within the domain of their tandem repeats (Ecker *et al.*, 2006).

Given that the LLA1271 proteins are non-covalently bound to the exine wall and the observation of distorted exine formation in *TAP::LLA1271* pollen, LLA1271 is thus thought to be required for primordial tectum formation or exine patterning, similar to the type 3 *kaonashi* (*kns*) mutants (Suzuki *et al.*, 2008) and unlike other identified proteins involved in the biosynthesis of sporopollenin precursors in the tapetum (Yi *et al.*, 2010; Chen *et al.*, 2011; Sun *et al.*, 2013). Suzuki *et al.* (2008) hypothesized that the type 3 *kns* mutants are involved in the biosynthesis or deposition of sporopollenin on a growing tectum. The abnormal distribution of baculae may also contribute to the phenotypes of the type 3 *kns* mutants (Dobritsa *et al.*, 2011). The pollen exine contains two layers, the inner endexine and the outer ectexine. Through the elaboration of columellae and tectum, the outer ectexine forms a complex layer providing most of the species-specific variation in pollen wall patterning. Although the function of LLA1271 protein is not clear, the observation that the protein is distributed only in the exine of developing microspores and the appearance of distorted exine formation suggest that LLA1271 is required for pollen wall integrity and exine patterning. Additionally, *TAP::LLA1271* plants exhibit impaired stamen and low pollen germination. Given that the *LLA1271* mRNA was not detected in the filament (Fig. 3A), it is not easy to elucidate the appearance of the short stamen in *TAP::LLA1271* for the present. Nevertheless, hormones such as auxin, GA, brassinosteroids, and jasmonic acid have been reportedly involved in the regulation of filament growth (Cheng *et al.*, 2009; Ye *et al.*, 2010; Chae *et al.*, 2012).

A novel anther-specific gene has been identified whose encoded proteins may represent novel adhesin-like proteins in lily anthers. The proteins synthesized both in the tapetum and in the microspore are secreted and deposited in the exine layer of microspores before the occurrence of microspore mitosis. Thus, this work suggests that the protein may be associated with exine formation during microspore development and that the *LLA1271* gene can be used as a molecular marker for the response of anthers to GA. Isolation of tapetal genes that are GA responsive is an important step towards understanding the role of GA in plant reproduction.

## Supplementary data

Supplementary data are available at *JXB* online.

Figure S1. Nucleotide and predicted amino acid sequences of *LLA1271* cDNA clones.

Figure S2. Identification of two forms of *LLA1271*.

Figure S3. RT–PCR analysis of *TAP::LLA1271* transgenic lines.

Figure S4. Transmission electron micrographs of *TAP:: LLA1271* pollen grains.

Supplementary Data

## Abbreviations:

DIG: digoxigenin
2D-PAGE: two-dimensional polyacrylamide gel electrophoresis GA, gibberellin
LLA: *Lilium longiflorum* anther
MOPS: 3-*N*-morpholino propanesulphonic acid
NBD: 2,5-norbornadien
RACE PCR: rapid amplification of cDNA ends-polymerase chain reaction
SEM: scanning electron microscopy
TEM: transmission electron microscopy.
