# Supplementary material for: A novel lily anther-specific gene encodes adhesin-like proteins associated with exine formation during anther development
Source: J Exp Bot. 2014 Mar 3;65(8):2023–37. doi: 10.1093/jxb/eru051 (PMC3991738; doi:10.1093/jxb/eru051)
Supplement: Supplementary Data [file supp_65_8_2023__index.html]

A novel lily anther-specific gene encodes adhesin-like proteins associated with exine formation during anther development — A novel lily anther-specific gene encodes adhesin-like proteins associated with exine formation during anther development — Supplementary Data 

# A novel lily anther-specific gene encodes adhesin-like proteins associated with exine formation during anther development

## Supplementary Data

Data files

**Files in this Data Supplement:**

- Supplementary Data - Supplementary Data
