# Supplementary material for: A novel lily anther-specific gene encodes adhesin-like proteins associated with exine formation during anther development
Source: J Exp Bot. 2014 Mar 3;65(8):2023–37. doi: 10.1093/jxb/eru051 (PMC3991738; doi:10.1093/jxb/eru051)
Supplement: Supplementary Data [file supp_eru051_jexbot107532_file001.pdf]

A novel lily anther-specific gene encodes adhesin-like proteins associated with exine formation during anther development. *Ming-Che Liu, Cheng-Shou Yang, Fang-Ling Yeh, Chi-Hsuan Wei, Wann-Neng Jane, Mei-Chu Chung and Co-Shine Wang*

SUPPLEMENTARY DATA

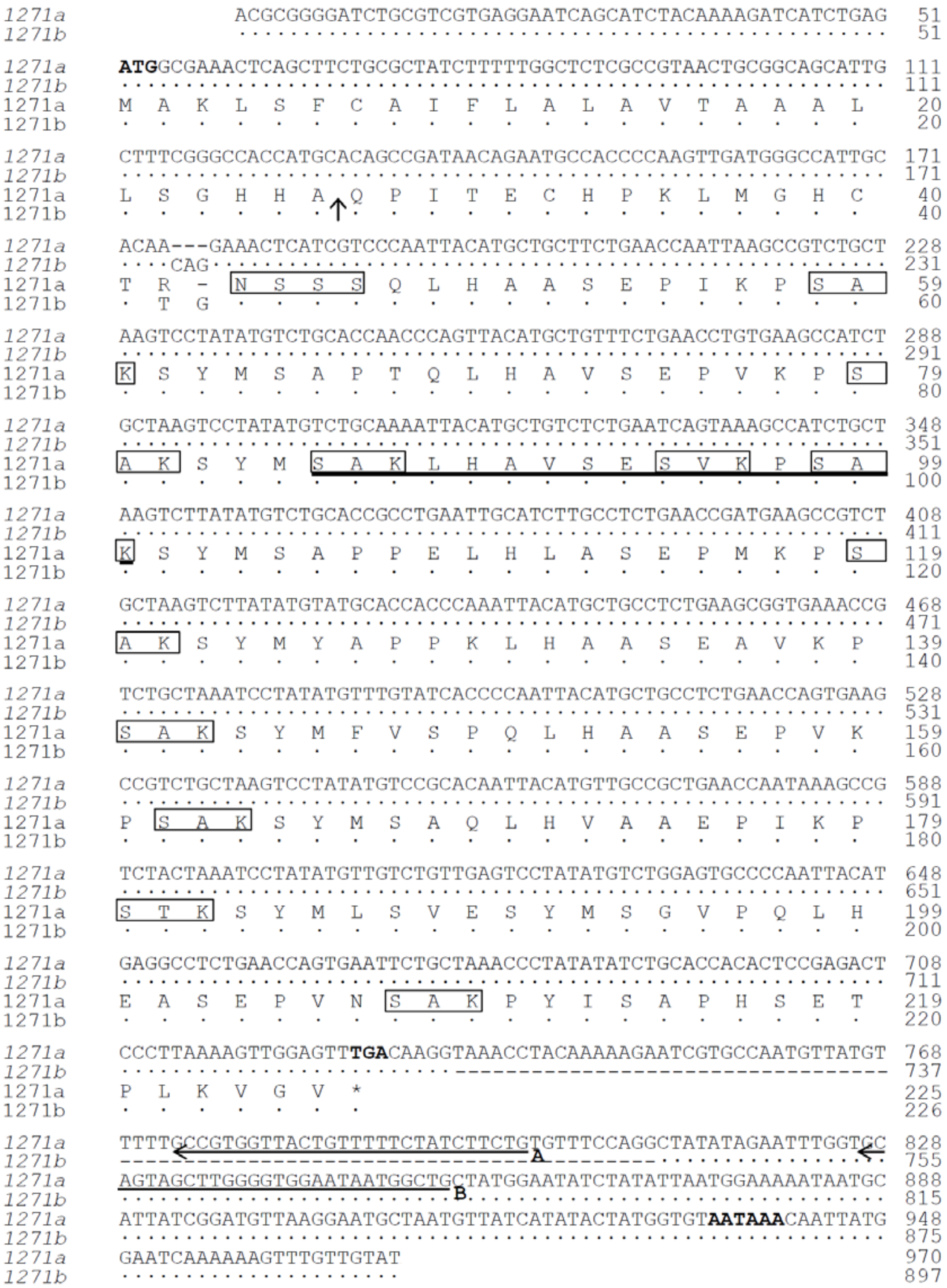

Supplementary Fig. S1. Nucleotide and predicted amino acid sequences of *LLA1271* cDNA clones. Bold letters in the nucleotide sequence indicate the start and stop codons and the polyadenylation signal. A vertical arrow indicates the cut site of a signal peptide of LLA1271 proteins in the N-terminus. The underlined sequence indicates the synthetic peptide used for the production of antiserum. The box indicates the putative N-glycosylation (N-X-S/T) and phosphorylation (S/T-X-K/R) sites. 3´-primer A and B used for 5´-RACE are also indicated. A dash in the sequence indicates a gap introduced in order to maintain good alignment. Identities are represented by dots.

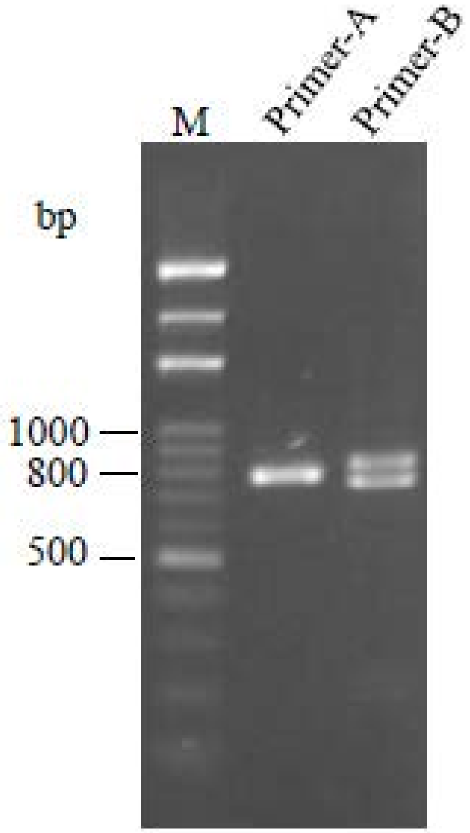

Supplementary Fig. S2. Identification of two forms of *LLA1271*. 5'-RACE PCR was performed with primers A and B, respectively. The PCR products were fractioned by 1.5% agarose gel and stained with EtBr. The 1 kb ladder markers (M) are indicated at the left.

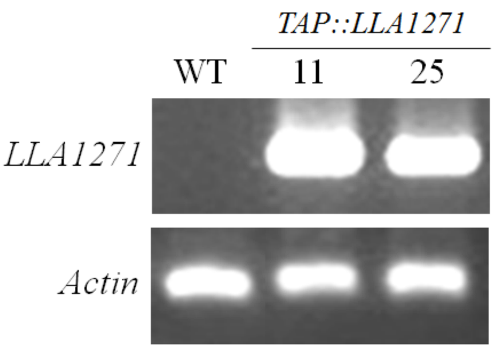

Supplementary Fig. S3. RT-PCR analysis of *TAP::LLA1271* transgenic lines. RT-PCR was performed on total RNA (1 µg/line) isolated from five week-old inflorescence of wild-type and the two *TAP::LLA1271* transgenic lines 11 and 25. The fragment of *LLA1271* was amplified using a pair of specific primers to *LLA1271*. The *actin* gene was used as a quantitative control.

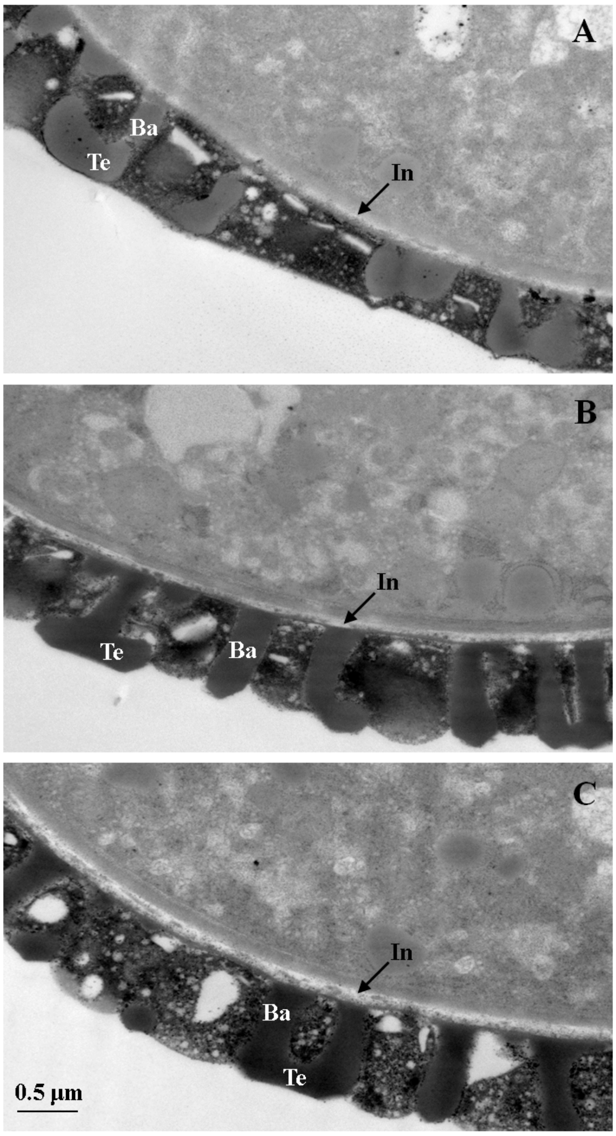

Supplementary Fig. S4. Transmission electron micrographs of *TAP::LLA1271* pollen grains. Micrographs of the pollen wall regions of the two *TAP::LLA1271* transgenic lines 11 (B) and 25 (C) were compared with that of wild-type pollen wall (A). Ba, Bacula; In, intine; Te, tectum.
